# Supplementary material for: In vitro and in silico pharmaco-nutritional assessments of some lesser-known Nigerian nuts: Persea americana, Tetracarpidium conophorum, and Terminalia catappa
Source: PLoS One. 2025 Apr 9;20(4):e0319756. doi: 10.1371/journal.pone.0319756 (PMC11981145; doi:10.1371/journal.pone.0319756)
Supplement: S1 Raw Data — (ZIP) [file pone.0319756.s001.zip › Raw data/Almond Library Search Report_113351.pdf]

Library Search Report

Data Path : D:\MassHunter\GCMS\1\DATA\
Data File : Phytochemical 27.D
Acq On : 09 Mar 2022 15:04
Operator : Justin
Sample : Almond
Misc :
ALS Vial : 1 Sample Multiplier: 1

Search Libraries: D:\MassHunter\Library\NIST14.L Minimum Quality: 0

Unknown Spectrum: Apex
Integration Events: ChemStation Integrator - autoint1.e

Pk#	RT	Area%	Library/ID	Ref#	CAS#	Qual
1	0.514	0.59	D:\MassHunter\Library\NIST14.L			
			Decane, 2,2,4-trimethyl-	51456	062237-98-3	25
			Hexane, 2,2,4-trimethyl-	12986	016747-26-5	25
			2-Ethylhexanal	12721	1000411-44-7	10
2	0.555	0.42	D:\MassHunter\Library\NIST14.L			
			11-Tridecenyl propionate	115323	1000130-96-7	9
			Octane, 3-ethyl-	19652	005881-17-4	9
			Butyl isobutyl carbonate	43527	178442-31-4	9
3	0.605	1.24	D:\MassHunter\Library\NIST14.L			
			Azetidine, 1,2-dimethyl-	1601	051764-32-0	38
			2-Butene, 1-methoxy-, (Z)-	1820	010034-16-9	9
			1,2,3-Trimethyldiaziridine	1643	113604-56-1	9
4	0.631	2.42	D:\MassHunter\Library\NIST14.L			
			Pentane, 3-methyl-	1847	000096-14-0	45
			Pentane, 2,3-dimethyl-	4048	000565-59-3	9
			Pentane, 2,3-dimethyl-	4050	000565-59-3	9
5	0.669	4.15	D:\MassHunter\Library\NIST14.L			
			Pentane, 3-methyl-	1847	000096-14-0	9
			1-Ethylcyclopropanol	1787	057872-31-8	7
			Borinic acid, diethyl-	1657	004426-31-7	7
6	0.765	6.99	D:\MassHunter\Library\NIST14.L			
			Cyclopentane, methyl-	1512	000096-37-7	80
			1-Octene, 7-methyl-	11763	013151-06-9	64
			Cyclohexane	1473	000110-82-7	56
7	0.934	12.76	D:\MassHunter\Library\NIST14.L			
			Oxirane, (1-methylbutyl)-	7698	053229-39-3	50
			1-Pentene, 2-methyl-	1506	000763-29-1	43
			1-Hexene	1470	000592-41-6	43
8	1.014	5.39	D:\MassHunter\Library\NIST14.L			
			Cyclopentane, 1,3-dimethyl-	3443	002453-00-1	80
			1-Pentanol, 3,4-dimethyl-	8529	006570-87-2	72
			Cyclopentane, 1,3-dimethyl-, cis-	3456	002532-58-3	64

9	1.063	11.00	D:\MassHunter\Library\NIST14.L			
			Cyclopentane, 1,2-dimethyl-	3440	002452-99-5	58
			Cyclopentane, 1,2-dimethyl-, cis-	3457	001192-18-3	53
			Cyclopentane, 1,2-dimethyl-, trans	3467	000822-50-4	50
10	1.268	25.13	D:\MassHunter\Library\NIST14.L			
			Cyclohexane, methyl-	3389	000108-87-2	72
			Cyclopentane, 1-ethyl-3-methyl-, c<br>is-	6947	002613-66-3	59
			Cyclohexane, methyl-	3387	000108-87-2	53
11	1.581	0.57	D:\MassHunter\Library\NIST14.L			
			Hexane, 3-ethyl-4-methyl-	13007	003074-77-9	72
			Hexane, 3-ethyl-4-methyl-	13005	003074-77-9	64
			Carbonic acid, isobutyl 2-ethylhex<br>yl ester	93133	1000357-82-9	59
12	1.760	13.38	D:\MassHunter\Library\NIST14.L			
			Cyclohexane, 1,3-dimethyl-, trans-	6943	002207-03-6	64
			Cyclohexane, 1,3-dimethyl-, trans-	6944	002207-03-6	64
			Cyclohexane, 1,2-dimethyl-, trans-	6937	006876-23-9	58
13	1.988	4.74	D:\MassHunter\Library\NIST14.L			
			1H-Pyrazol-4-amine, 3-methyl-	2988	1000338-28-2	35
			Cyclohexane, 1,2-dimethyl-, trans-	6937	006876-23-9	30
			Octane	7760	000111-65-9	27
14	2.055	2.19	D:\MassHunter\Library\NIST14.L			
			Cyclohexane, 1,2-dimethyl- (cis/tr<br>ans)	6951	000583-57-3	60
			Cyclohexane, 1,2-dimethyl- (cis/tr<br>ans)	6950	000583-57-3	45
			Cyclohexane, 1,2-dimethyl-, trans-	6937	006876-23-9	45
15	2.309	0.15	D:\MassHunter\Library\NIST14.L			
			1,3-Cyclohexanediamine	7480	003385-21-5	43
			Heptane, 3,4-dimethyl-	12958	000922-28-1	38
			1,3-Cyclohexanediamine	7477	003385-21-5	38
16	2.466	0.95	D:\MassHunter\Library\NIST14.L			
			3,4-Dimethyl-2-hexene	6810	002213-37-8	53
			3,4-Dimethyl-2-hexene	6819	002213-37-8	49
			Cyclohexane, ethyl-	6787	001678-91-7	49
17	2.665	0.25	D:\MassHunter\Library\NIST14.L			
			Cyclohexane, 1,1,2-trimethyl-	11817	007094-26-0	53
			2,3-Dimethyl-3-heptene	11780	1000113-49-3	53
			Cyclopentane, 1-ethyl-3-methyl-, t<br>rans-	6953	002613-65-2	47
18	2.834	0.09	D:\MassHunter\Library\NIST14.L			
			Octane, 3,4-dimethyl-	19671	015869-92-8	43
			Dichloroacetic acid, 4-methylpenty<br>l ester	76809	1000282-43-7	43
			Cycloheptanone	6596	000502-42-1	38
19	2.949	0.17	D:\MassHunter\Library\NIST14.L			

```
                 Isobutyl nonyl carbonate           105956 959311-27-4 40
                 Hexane, 3-ethyl-4-methyl-           13005 003074-77-9 38
                 Carbonic acid, butyl nonyl ester   105961 1000314-63-6 37

20   3.064  0.93 D:\MassHunter\Library\NIST14.L
                 p-Xylene                             5162 000106-42-3 95
                 o-Xylene                             5168 000095-47-6 95
                 o-Xylene                             5170 000095-47-6 94

21   3.241  0.70 D:\MassHunter\Library\NIST14.L
                 o-Xylene                             5168 000095-47-6 95
                 o-Xylene                             5170 000095-47-6 94
                 p-Xylene                             5161 000106-42-3 94

22   3.437  0.49 D:\MassHunter\Library\NIST14.L
                 p-Xylene                             5162 000106-42-3 93
                 p-Xylene                             5159 000106-42-3 93
                 Benzene, 1,3-dimethyl-               5190 000108-38-3 93

23   3.792  0.07 D:\MassHunter\Library\NIST14.L
                 Cyclohexanepropanol-                20490 001124-63-6 27
                 Cyclohexanepropanol-                20481 001124-63-6 27
                 1,3-Cyclopentadiene, 5-(1-methylet   5202 002175-91-9 25
                 hylidene)-

24   4.027  0.17 D:\MassHunter\Library\NIST14.L
                 Chloroacetic acid, 6-chlorohexyl e  76808 1000330-85-5 50
                 ster
                 Cyclohexanemethanol                  7619 000100-49-2 35
                 Cyclohexanemethanol                  7618 000100-49-2 35

25   4.522  0.02 D:\MassHunter\Library\NIST14.L
                 2H-Pyran, 5,6-dihydro-2-methyl-      3316 055230-25-6 46
                 (E)-2-Butenylcyclopropane            2936 076588-98-2 43
                 1,5-Heptadiene, (Z)-                 2898 007736-34-7 43

26   4.573  0.02 D:\MassHunter\Library\NIST14.L
                 Carbonic acid, butyl nonyl ester   105961 1000314-63-6 53
                 Dodecyl isobutyl carbonate         146242 959067-22-2 53
                 n-Tetracosanol-1                   209169 000506-51-4 50

27   4.703  0.05 D:\MassHunter\Library\NIST14.L
                 1,3,5-Cycloheptatriene, 7-ethyl-     9622 017634-51-4 76
                 Benzene, propyl-                     9586 000103-65-1 53
                 Benzene, 1-ethyl-3-methyl-           9607 000620-14-4 49

28   4.841  0.09 D:\MassHunter\Library\NIST14.L
                 Benzene, 1-ethyl-4-methyl-           9610 000622-96-8 93
                 Benzene, 1-ethyl-3-methyl-           9609 000620-14-4 90
                 Benzene, 1-ethyl-3-methyl-           9604 000620-14-4 83

29   4.949  0.03 D:\MassHunter\Library\NIST14.L
                 Benzene, 1,2,4-trimethyl-            9602 000095-63-6 90
                 Mesitylene                           9581 000108-67-8 89
                 Benzene, 1-ethyl-3-methyl-           9609 000620-14-4 86

30   5.156  0.01 D:\MassHunter\Library\NIST14.L
```

```
                Benzene, 1-ethyl-3-methyl-           9604 000620-14-4 59
                Benzene, 1-ethyl-4-methyl-           9610 000622-96-8 53
                Benzene, 1-ethyl-2-methyl-           9608 000611-14-3 49

31   5.385  0.09 D:\MassHunter\Library\NIST14.L
                Benzene, 1,2,4-trimethyl-            9602 000095-63-6 91
                Benzene, 1,2,4-trimethyl-            9590 000095-63-6 91
                Benzene, 1,2,3-trimethyl-            9595 000526-73-8 87

32   5.917  0.02 D:\MassHunter\Library\NIST14.L
                2,4-Nonadiyne                        9583 063621-15-8 94
                1-Hexen-4-yne, 3-ethylidene-2-meth   9632 076003-39-9 70
                yl-
                Benzene, 1-ethyl-3-methyl-           9609 000620-14-4 58

33   5.983  0.00 D:\MassHunter\Library\NIST14.L
                Cyclohexane, 2-propenyl-            10865 002114-42-3 64
                5-Hepten-2-one, 4,6-dimethyl-       19201 031162-48-8 53
                Cyclohexane, tetradecyl-           140282 001795-18-2 47

34   6.434  0.01 D:\MassHunter\Library\NIST14.L
                Benzenemethanethiol, .alpha.-methy  17883 006263-65-6 45
                l-
                Benzeneacetaldehyde, .alpha.-methy  15709 000093-53-8 35
                l-
                Benzeneacetaldehyde, .alpha.-methy  15708 000093-53-8 30
                l-

35   6.566  0.01 D:\MassHunter\Library\NIST14.L
                Benzene, 4-ethyl-1,2-dimethyl-      15224 000934-80-5 58
                Benzene, 1-ethyl-2,3-dimethyl-      15216 000933-98-2 58
                Benzene, 2-ethyl-1,4-dimethyl-      15226 001758-88-9 53

36   7.239  0.00 D:\MassHunter\Library\NIST14.L
                Oxalic acid, isobutyl nonyl ester  132408 1000309-37-4 74
                Oxalic acid, cyclobutyl nonyl este 130368 1000309-70-0 47
                Ether, heptyl hexyl                 65237 007289-40-9 47

37   7.288  0.00 D:\MassHunter\Library\NIST14.L
                Ether, hexyl pentyl                 41574 032357-83-8 40
                Borinic acid, diethyl-               1658 004426-31-7  9
                Undecane, 2,4-dimethyl-             51440 017312-80-0  9

38  21.433  0.04 D:\MassHunter\Library\NIST14.L
                10-Undecyn-1-ol                     38134 002774-84-7 43
                1,4-Cyclooctadiene, (Z,Z)-           5550 016327-22-3 38
                Butyraldehyde, 4-(methylenecyclopr  10782 1000156-86-5 35
                opyl)-

39  21.823  4.57 D:\MassHunter\Library\NIST14.L
                9,12,15-Octadecatrien-1-ol, (Z,Z,Z 125005 000506-44-5 94
                )-
                Methyl 8,11,14-heptadecatrienoate  138417 1000336-35-1 93
                9,12,15-Octadecatrienoic acid, (Z, 138418 000463-40-1 91
                Z,Z)-

40  23.171  0.00 D:\MassHunter\Library\NIST14.L
```

```
                4-Decyne                              17243 002384-86-3 14
                4-Octyne                               6001 001942-45-6 12
                1,3,7-Octatriene                       5501 001002-35-3 10

 41  23.261  0.01 D:\MassHunter\Library\NIST14.L
                Acetic acid, [4-(1-hydroxy-1-methy  76094 1000197-22-2 50
                lethyl)cyclohex-1-enyl]methyl este
                trans-3-Cyclopropyl-7-(2-methoxyet  59781 1000223-15-8 43
                hyl)norcarane
                Cyclohexene, 3-ethenyl-                5529 000766-03-0 42

 42  24.565  0.00 D:\MassHunter\Library\NIST14.L
                Didodecyl phthalate                  266710 002432-90-8 53
                Phthalic acid, monooctyl ester       138067 005393-19-1 53
                Phthalic acid, monodecyl ester       165194 024539-60-4 53

 43  25.634  0.01 D:\MassHunter\Library\NIST14.L
                3-Octyne                               5993 015232-76-5 50
                5-Dodecyne                            36560 019780-12-2 50
                Undec-10-ynoic acid, tridec-2-yn-1 213761 1000406-96-9 47
                -yl ester

 44  25.738  0.05 D:\MassHunter\Library\NIST14.L
                1,4,9-Decatriene, (Z)-                16101 1000155-93-2 76
                cis,cis,cis-7,10,13-Hexadecatriena  96798 056797-43-4 74
                3-Decen-1-yne, (E)-                   16085 002807-10-5 46

 45  25.783  0.01 D:\MassHunter\Library\NIST14.L
                Bicyclo[5.1.0]oct-3-ene                5522 000659-84-7 55
                2,7-Octadien-1-ol                     11569 023578-51-0 43
                Bicyclo[4.1.1]oct-2-ene                5523 016544-26-6 38

 46  28.388  0.01 D:\MassHunter\Library\NIST14.L
                .gamma.-Tocopherol                   245804 007616-22-0 64
                .gamma.-Tocopherol                   245806 007616-22-0 64
                .gamma.-Tocopherol                   245805 007616-22-0 53
```
