# Supplementary material for: In vitro and in silico pharmaco-nutritional assessments of some lesser-known Nigerian nuts: Persea americana, Tetracarpidium conophorum, and Terminalia catappa
Source: PLoS One. 2025 Apr 9;20(4):e0319756. doi: 10.1371/journal.pone.0319756 (PMC11981145; doi:10.1371/journal.pone.0319756)

Area Percent Report

Data Path : D:\MassHunter\GCMS\1\data\
Data File : Phytochemical 19.D
Acq On : 09 Mar 2022 12:22
Operator : justin
Sample : avocado
Misc :
ALS Vial : 1 Sample Multiplier: 1

Integration Parameters: rteint.p
Integrator: RTE
Smoothing : ON Filtering: 5
Sampling : 1 Min Area: 0.5 % of largest Peak
Start Thrs: 0.2 Max Peaks: 100
Stop Thrs : 0 Peak Location: CENTROID

If leading or trailing edge < 100 prefer < Baseline drop else tangent >
Peak separation: 5

Method : D:\MassHunter\GCMS\1\methods\Phytochemical screening3.M
Title :

Signal : TIC: Phytochemical 19.D\data.ms

peak #	R.T. min	first scan	max scan	last scan	PK TY	peak height	corr. area	corr. % max.	% of total
1	0.191	8	11	14	rBV	292671	298129	28.67%	12.169%
2	0.266	23	24	28	rVB3	13064	10101	0.97%	0.412%
3	0.313	28	33	45	rVB	23187	38114	3.66%	1.556%
4	0.434	47	54	57	rBV3	467930	1039960	100.00%	42.451%
5	0.539	69	72	74	rBV2	98643	117116	11.26%	4.781%
6	0.649	89	91	99	rVB2	845233	828590	79.68%	33.823%
7	0.915	131	138	142	rBV3	17941	27271	2.62%	1.113%
8	0.969	142	147	160	rVB4	9320	23778	2.29%	0.971%
9	23.068	3998	4009	4016	rBB4	5333	11883	1.14%	0.485%
10	23.378	4051	4063	4071	rBB4	15084	33703	3.24%	1.376%
11	23.854	4137	4147	4150	rBV3	3979	7478	0.72%	0.305%
12	23.889	4150	4153	4161	rVB3	4869	7173	0.69%	0.293%
13	24.133	4187	4195	4202	rBB3	4249	6512	0.63%	0.266%

Sum of corrected areas: 2449808

Phytochemic...screening3.M Wed Mar 09 13:13:48 2022

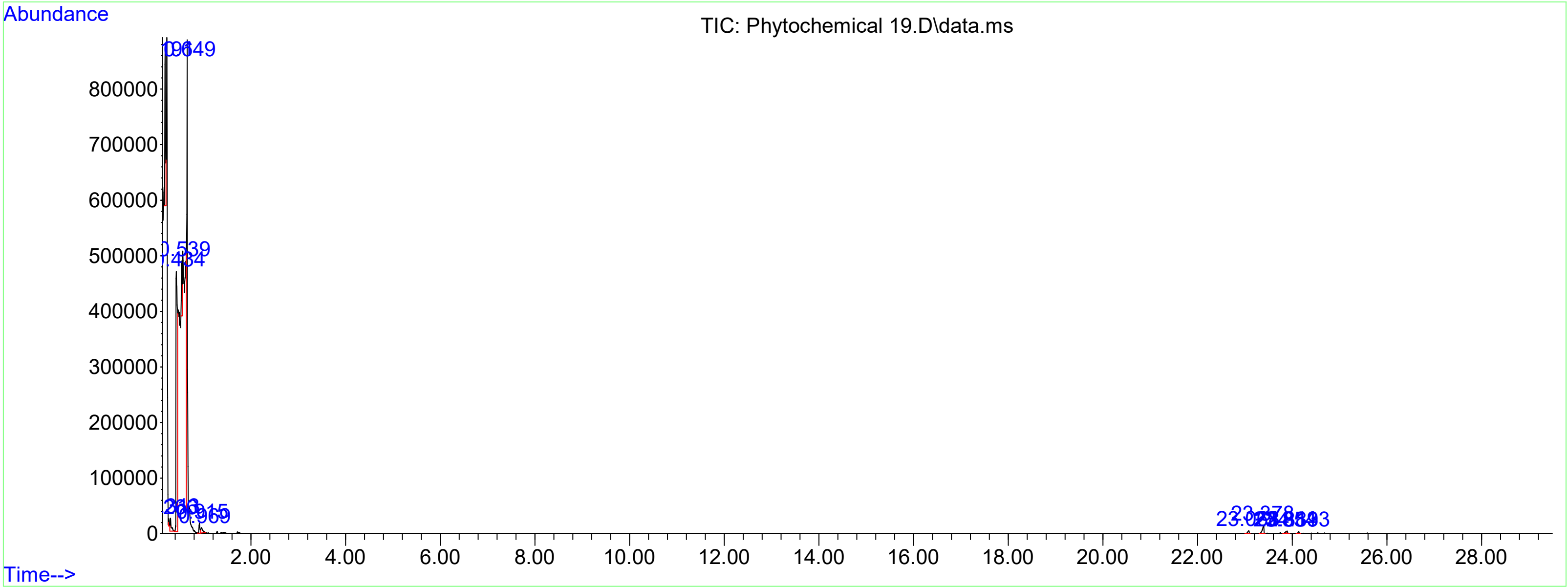

Supplement: S1 Raw Data — (ZIP) [file pone.0319756.s001.zip › Raw data/Avocado chromatogram with percentage report_113507.pdf]
