# Supplementary material for: In vitro and in silico pharmaco-nutritional assessments of some lesser-known Nigerian nuts: Persea americana, Tetracarpidium conophorum, and Terminalia catappa
Source: PLoS One. 2025 Apr 9;20(4):e0319756. doi: 10.1371/journal.pone.0319756 (PMC11981145; doi:10.1371/journal.pone.0319756)
Supplement: S1 Raw Data — (ZIP) [file pone.0319756.s001.zip › Raw data/Avocado Library Search Report Real_113509.pdf]

Library Search Report

Data Path : D:\MassHunter\GCMS\1\data\
Data File : Phytochemical 19.D
Acq On : 09 Mar 2022 12:22
Operator : justin
Sample : avocado
Misc :
ALS Vial : 1 Sample Multiplier: 1

Search Libraries: D:\MassHunter\Library\NIST14.L Minimum Quality: 0

Unknown Spectrum: Apex
Integration Events: RTE Integrator - rteint.p

Pk#	RT	Area%	Library/ID	Ref#	CAS#	Qual
1	0.191	12.17	D:\MassHunter\Library\NIST14.L			
			Methylene chloride	1543	000075-09-2	64
			Methylene chloride	1545	000075-09-2	64
			Methane-d, trichloro-	9324	000865-49-6	52
2	0.266	0.41	D:\MassHunter\Library\NIST14.L			
			Hexane, 2,2,3-trimethyl-	12999	016747-25-4	47
			Cyanic acid, 2-methylpropyl ester	3575	001768-25-8	43
			Cyclohexane	1473	000110-82-7	43
3	0.313	1.56	D:\MassHunter\Library\NIST14.L			
			1-Hexene, 3,4-dimethyl-	6835	016745-94-1	17
			Azetidine, 2-methyl-	621	019812-49-8	16
			Cyclohexane	1475	000110-82-7	9
4	0.434	42.45	D:\MassHunter\Library\NIST14.L			
			2-[2-(2-Chloro-ethoxy)-ethoxy]-phe nol	79210	1000317-46-1	59
			Propanoic acid, 2-chloro-, methyl ester, (S)-	9941	073246-45-4	59
			Propanoic acid, 2-chloro-, methyl ester, (R)-	9940	077287-29-7	59
5	0.539	4.78	D:\MassHunter\Library\NIST14.L			
			2-[2-(2-Chloro-ethoxy)-ethoxy]-phe nol	79210	1000317-46-1	45
			2-Chloroethyl methyl sulfone	19773	050890-51-2	45
			Propanoic acid, 2-chloro-, methyl ester	9934	017639-93-9	37
6	0.649	33.82	D:\MassHunter\Library\NIST14.L			
			Propanoic acid, 2-chloro-, methyl ester, (R)-	9940	077287-29-7	47
			Ethane, 1,1-dichloro-	3055	000075-34-3	47
			Propanoic acid, 2-chloro-, methyl ester	9934	017639-93-9	47
7	0.915	1.11	D:\MassHunter\Library\NIST14.L			
			2-Heptene	3344	000592-77-8	58
			3-Heptene, (E)-	3360	014686-14-7	58
			Isopropylcyclobutane	3385	000872-56-0	53
8	0.969	0.97	D:\MassHunter\Library\NIST14.L			
			Propanedinitrile, methylene-	1024	000922-64-5	40
			1,5-Hexadiyne	1031	000628-16-0	25
			1,5-Hexadiyne	1029	000628-16-0	12
9	23.068	0.49	D:\MassHunter\Library\NIST14.L			
			3-Pyrrolidinol	1890	040499-83-0	40
			2-Butanone, oxime	1894	000096-29-7	4
			2-Butanone, oxime	1895	000096-29-7	4
10	23.378	1.38	D:\MassHunter\Library\NIST14.L			
			3-Butenenitrile, 2-methyl-	1141	016529-56-9	38

Data Path : D:\MassHunter\GCMS\1\data\
Data File : Phytochemical 19.D
Acq On : 09 Mar 2022 12:22
Operator : justin
Sample : avocado
Misc :
ALS Vial : 1 Sample Multiplier: 1

Search Libraries: D:\MassHunter\Library\NIST14.L Minimum Quality: 0

Unknown Spectrum: Apex
Integration Events: RTE Integrator - rteint.p

Pk#	RT	Area%	Library/ID	Ref#	CAS#	Qual
			2-Methyl-2-butenenitrile	1140	004403-61-6	38
			10-Azido-1-decanethiol	78716	1152113-44-4	9
11	23.854	0.31	D:\MassHunter\Library\NIST14.L			
			Succinimide, thio-	7847	004166-00-1	4
			2,5-Pyrrolidinedione, 1-hydroxy-	7844	006066-82-6	4
			2,5-Pyrrolidinedione, 1-hydroxy-	7845	006066-82-6	4
12	23.889	0.29	D:\MassHunter\Library\NIST14.L			
			3-Oxo-hexanedioic acid, 1-benzyl e ster	111178	071010-31-6	9
			Benzeneethanamine, .alpha.,2,6-tri methyl-, (.+/-.)-	34200	057204-69-0	7
			2-Pentenal, 5-phenyl-	31935	033046-84-3	4
13	24.133	0.27	D:\MassHunter\Library\NIST14.L			
			Pent-2-ynal	1174	055136-52-2	9
			Pent-2-ynal	1175	055136-52-2	9
			4-Heptanol, 2-methyl-	14003	021570-35-4	9
