# Supplementary material for: In vitro and in silico pharmaco-nutritional assessments of some lesser-known Nigerian nuts: Persea americana, Tetracarpidium conophorum, and Terminalia catappa
Source: PLoS One. 2025 Apr 9;20(4):e0319756. doi: 10.1371/journal.pone.0319756 (PMC11981145; doi:10.1371/journal.pone.0319756)
Supplement: S1 Raw Data — (ZIP) [file pone.0319756.s001.zip › Raw data/phytochemical analysis result_113510.docx]

DETERMINATION OF PHYTOCHEMICALS IN 3 SAMPLES

| **S/N** | **Name of compound** | **Percentage concentration (%)** | **Concentration in ppm** | **Phytochemical group** |
| --- | --- | --- | --- | --- |
| **ALMOND** | | | | Alkaloids |
| **1** | Azetidine, 1,2-dimethyl- | 1.23 | 2.113 |  |
|  | 1,2,3-Trimethyldiaziridine | 1.00 | 1.720 |  |
|  | 1H-Pyrazol-4-amine, 3-methyl- | 3.79 | 6.518 |  |
|  | 2H-Pyran, 5,6-dihydro-2-methyl- | 0.01 | 0.017 |  |
|  | Didodecyl phthalate | 0.02 | 0.034 |  |
|  | Phthalic acid, monooctyl ester | 0.1 | 0.172 |  |
| **AVOCADO** | | | |  |
|  |  |  |  | Alkaloids |
| **1** | 2,5-Pyrrolidinedione, 1-hydroxy- | 0.21 | 0.359 |  |
|  | Azetidine, 2-methyl- | 0.11 | 0.188 |  |
|  | Succinimide, thio- | 0.21 | 0.359 |  |
|  | 10-Azido-1-decanethiol | 0.31 | 0.530 |  |
| **WALNUT** | | | | |
| **1** | Tert-butyl[(cyanomethyl)-oxo-$l^{5  }-azanylidene]amine | 0.05 | 0.072 | Alkaloids |
|  | 1,2,3-Trimethyldiaziridine | 0.76 | 1.094 |  |
|  | 1,2-Dimethylaziridine | 0.73 | 1.051 |  |
|  | 3-Aminopyrrolidine | 2.11 | 3.038 |  |
|  | Fumaronitrile | 2.11 | 3.038 |  |
|  | 1H-Imidazole, 4,5-dihydro-2,4-dimethyl- | 12.53 | 18.04 |  |
|  | Aziridine, 2,2-dimethyl- | 1.22 | 1.757 |  |
|  | Piperidine | 0.0051 | 0.0073 |  |
|  | Diaziridine, 3-ethyl-3-methyl- | 0.0001 | 0.00014 |  |
| **2** | Mesitylene | 0.30 | 0.432 | Flavonoid |

Yours Faithfully


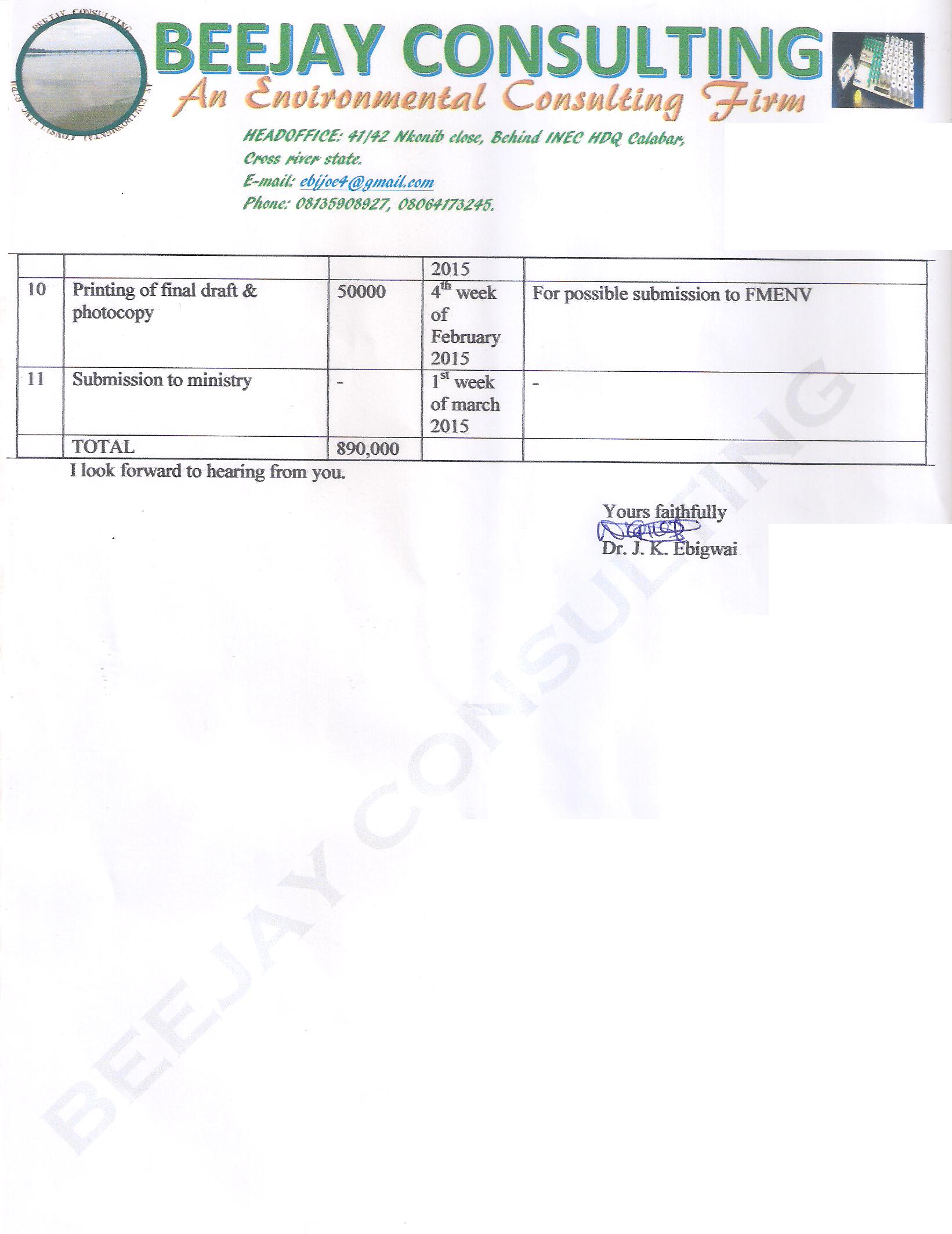


Prof. J.K. Ebigwai

**MD/CEO**
