# Supplementary material for: In vitro and in silico pharmaco-nutritional assessments of some lesser-known Nigerian nuts: Persea americana, Tetracarpidium conophorum, and Terminalia catappa
Source: PLoS One. 2025 Apr 9;20(4):e0319756. doi: 10.1371/journal.pone.0319756 (PMC11981145; doi:10.1371/journal.pone.0319756)

Area Percent Report

Data Path : D:\MassHunter\GCMS\1\DATA\
Data File : Phytochemical 28.D
Acq On : 09 Mar 2022 16:19
Operator : Justin
Sample : Walnut
Misc :
ALS Vial : 1 Sample Multiplier: 1

Integration Parameters: autoint1.e
Integrator: ChemStation

Method : D:\MassHunter\GCMS\1\methods\Phytochemical screening new.M
Title :

Signal : TIC: Phytochemical 28.D\data.ms

peak #	R.T. min	first scan	max scan	last scan	PK TY	peak height	corr. area	corr. % max.	% of total
1	0.478	48	63	70	BV 2	1228294	26811089	0.56%	0.103%
2	0.527	70	72	75	VV	1271136	10925832	0.23%	0.042%
3	0.594	75	84	87	VV	23613671	191976131	4.04%	0.738%
4	0.631	87	90	94	VV	30886391	361988903	7.61%	1.391%
5	0.670	94	97	101	VV 2	28383724	498406604	10.48%	1.915%
6	0.716	101	105	114	VV	22785926	629855435	13.24%	2.420%
7	0.788	114	118	122	VV	40430712	527102168	11.08%	2.025%
8	0.833	122	126	138	VV 2	39238102	994436358	20.91%	3.821%
9	0.967	138	149	152	VV 3	56477054	1638554718	34.45%	6.296%
10	1.007	152	156	164	VV 4	67234010	2307053886	48.50%	8.864%
11	1.082	164	169	175	VV 3	49369546	1444164395	30.36%	5.549%
12	1.148	175	181	188	VV 4	50150077	1766577507	37.14%	6.788%
13	1.243	188	197	202	VV	33567219	951774820	20.01%	3.657%
14	1.451	202	233	239	VV 6	65480191	4756842379	100.00%	18.277%
15	1.497	239	241	245	VV	13438932	145223817	3.05%	0.558%
16	1.539	245	249	253	VV	10884902	128345126	2.70%	0.493%
17	1.603	253	260	267	VV	3493148	50277716	1.06%	0.193%
18	1.694	267	276	280	VV 2	1986273	53008654	1.11%	0.204%
19	1.817	280	297	303	VV 4	56473048	2438438366	51.26%	9.369%
20	1.888	303	310	314	VV 6	70177423	2036085476	42.80%	7.823%
21	1.951	314	321	326	VV 4	52363410	1444322840	30.36%	5.549%
22	2.015	326	332	336	VV	5663810	89222315	1.88%	0.343%
23	2.161	336	358	364	VV 4	56658079	1664624894	34.99%	6.396%
24	2.224	364	369	374	VV	9440866	81601194	1.72%	0.314%
25	2.266	374	376	378	VV 2	182388	1880502	0.04%	0.007%
26	2.297	378	381	386	VV 2	212082	3428170	0.07%	0.013%
27	2.364	386	393	396	VV	95039	2279958	0.05%	0.009%
28	2.479	396	413	416	VV 3	328610	11715558	0.25%	0.045%
29	2.549	416	425	428	VV 2	6007281	99198466	2.09%	0.381%
30	2.589	428	432	449	VV 3	21230980	379700427	7.98%	1.459%
31	2.765	449	463	466	VV	176786	3628590	0.08%	0.014%
32	2.804	466	470	474	VV	345976	5163845	0.11%	0.020%
33	2.853	474	478	484	VV 2	81444	1724206	0.04%	0.007%
34	3.018	484	507	516	VV 2	1102735	33524613	0.70%	0.129%
35	3.212	516	541	549	VV 2	30465043	971756114	20.43%	3.734%
36	3.278	549	553	560	VV 2	137928	3054288	0.06%	0.012%
37	3.365	560	568	576	VV	386622	8430400	0.18%	0.032%
38	3.441	576	581	586	VV 3	48197	980074	0.02%	0.004%
39	3.557	586	601	610	VV 2	12710509	212352590	4.46%	0.816%
40	3.645	610	617	620	VV 2	98122	1456236	0.03%	0.006%
41	3.683	620	624	627	VV	174999	2114579	0.04%	0.008%
42	3.721	627	630	635	VV 3	40104	562184	0.01%	0.002%

Data Path : D:\MassHunter\GCMS\1\DATA\
Data File : Phytochemical 28.D
Acq On : 09 Mar 2022 16:19
Operator : Justin
Sample : Walnut
Misc :
ALS Vial : 1 Sample Multiplier: 1

Integration Parameters: autoint1.e
Integrator: ChemStation

Method : D:\MassHunter\GCMS\1\methods\Phytochemical screening new.M
Title :

43	3.794	635	643	650	VV 5	20557	569735	0.01%	0.002%
44	3.890	650	660	672	VV	274494	4419754	0.09%	0.017%
45	4.065	672	690	705	VV 3	236671	6760590	0.14%	0.026%
46	4.207	705	715	733	VV 5	37400	1595313	0.03%	0.006%
47	4.350	733	740	745	VV 4	20984	433579	0.01%	0.002%
48	4.407	745	750	753	VV 6	10037	175175	0.00%	0.001%
49	4.450	753	758	761	VV 4	14887	345056	0.01%	0.001%
50	4.509	761	768	771	VV 4	41347	815519	0.02%	0.003%
51	4.546	771	774	779	VV	64939	1136355	0.02%	0.004%
52	4.588	779	782	789	VV 5	30127	718383	0.02%	0.003%
53	4.656	789	794	796	VV 2	19680	338932	0.01%	0.001%
54	4.720	796	805	813	VV	205738	4778076	0.10%	0.018%
55	4.851	813	828	846	VV	244454	5063464	0.11%	0.019%
56	5.007	846	855	858	VV 3	18397	364932	0.01%	0.001%
57	5.055	858	863	875	VV 6	42970	1049080	0.02%	0.004%
58	5.155	875	881	889	VV 4	10983	240175	0.01%	0.001%
59	5.335	889	912	925	VV	866651	14388536	0.30%	0.055%
60	5.758	979	986	994	VV 3	19922	377899	0.01%	0.001%
61	5.886	994	1009	1016	PV 6	37321	1046409	0.02%	0.004%
62	5.967	1016	1023	1031	VV 3	15793	289757	0.01%	0.001%
63	6.437	1088	1105	1111	BV 2	29877	476021	0.01%	0.002%
64	6.579	1124	1130	1146	VV 4	12353	252991	0.01%	0.001%
65	7.305	1240	1257	1264	PV 2	18426	311284	0.01%	0.001%

Sum of corrected areas: 26026518442

Area Percent Report

Data Path : D:\MassHunter\GCMS\1\DATA\
Data File : Phytochemical 28.D
Acq On : 09 Mar 2022 16:19
Operator : Justin
Sample : Walnut
Misc :
ALS Vial : 1 Sample Multiplier: 1

Integration Parameters: autoint1.e
Integrator: ChemStation

Method : D:\MassHunter\GCMS\1\methods\Phytochemical screening new.M
Title :

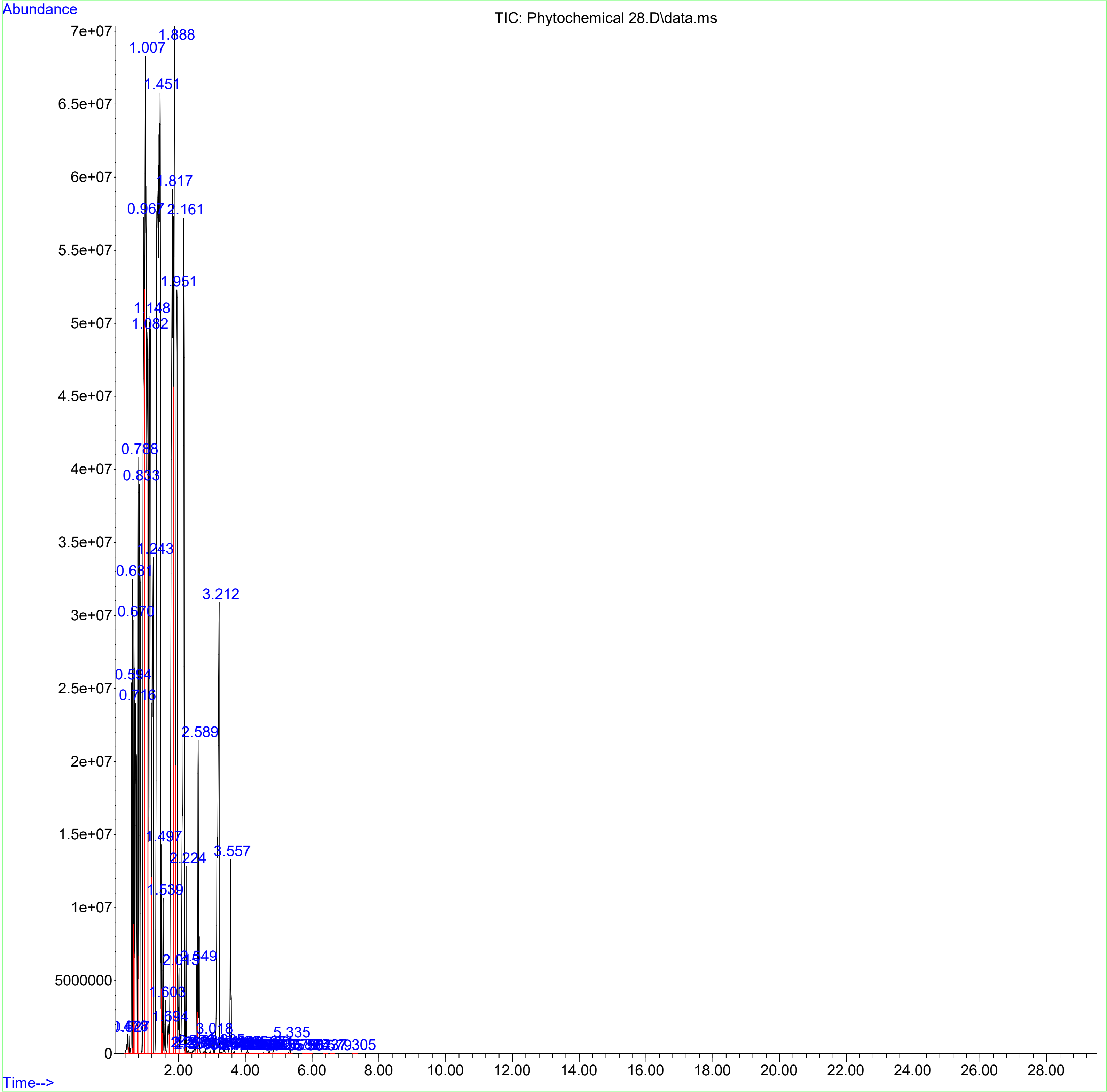

Supplement: S1 Raw Data — (ZIP) [file pone.0319756.s001.zip › Raw data/Walnut Chromatogram and Percentage Report_113514.pdf]
