# Supplementary material for: In vitro and in silico pharmaco-nutritional assessments of some lesser-known Nigerian nuts: Persea americana, Tetracarpidium conophorum, and Terminalia catappa
Source: PLoS One. 2025 Apr 9;20(4):e0319756. doi: 10.1371/journal.pone.0319756 (PMC11981145; doi:10.1371/journal.pone.0319756)
Supplement: S1 Raw Data — (ZIP) [file pone.0319756.s001.zip › Raw data/Walnut Library Search 1_113515.pdf]

Library Search Report

Data Path : D:\MassHunter\GCMS\1\DATA\
Data File : Phytochemical 28.D
Acq On : 09 Mar 2022 16:19
Operator : Justin
Sample : Walnut
Misc :
ALS Vial : 1 Sample Multiplier: 1

Search Libraries: D:\MassHunter\Library\NIST14.L Minimum Quality: 0

Unknown Spectrum: Apex
Integration Events: ChemStation Integrator - autoint1.e

Pk#	RT	Area%	Library/ID	Ref#	CAS#	Qual
1	0.478	0.10	D:\MassHunter\Library\NIST14.L			
			Butane, 1-chloro-2-methyl-	5109	000616-13-7	23
			Tert-butyl[(cyanomethyl)-oxo-$l^{5 }-azanylidene]amine	19348	1000411-42-0	9
			10-Azido-1-decanethiol	78716	1152113-44-4	9
2	0.527	0.04	D:\MassHunter\Library\NIST14.L			
			Butane, 2,2-dimethyl-	1856	000075-83-2	83
			Butane, 2,2-dimethyl-	1850	000075-83-2	83
			Butane, 2,2-dimethyl-	1855	000075-83-2	83
3	0.594	0.74	D:\MassHunter\Library\NIST14.L			
			Pentane, 2-methyl-	1844	000107-83-5	59
			Furan, tetrahydro-2-methyl-	1827	000096-47-9	53
			Furan, tetrahydro-2-methyl-	1826	000096-47-9	53
4	0.631	1.39	D:\MassHunter\Library\NIST14.L			
			1,2,3-Trimethyldiaziridine	1643	113604-56-1	59
			1,2-Dimethylaziridine	623	030757-96-1	50
			Hexane, 2,2,5,5-tetramethyl-	19718	001071-81-4	36
5	0.670	1.91	D:\MassHunter\Library\NIST14.L			
			n-Hexane	1842	000110-54-3	36
			2-Pentanone	1732	000107-87-9	10
			2-Ethyl-oxetane	1754	1000386-40-2	9
6	0.716	2.42	D:\MassHunter\Library\NIST14.L			
			n-Hexane	1842	000110-54-3	72
			2-Ethyl-oxetane	1754	1000386-40-2	50
			3-Aminopyrrolidine	1637	079286-79-6	9
7	0.788	2.03	D:\MassHunter\Library\NIST14.L			
			2-Pentene, 3-methyl-, (Z)-	1528	000922-62-3	52
			2-Pentene, 3-methyl-, (E)-	1530	000616-12-6	50
			Pentane, 3-methylene-	1513	000760-21-4	50
8	0.833	3.82	D:\MassHunter\Library\NIST14.L			
			1-Pentene, 3-methyl-	1497	000760-20-3	50
			2-Pentene, 3-methyl-, (Z)-	1532	000922-62-3	49
			2-Pentene, 3-methyl-, (Z)-	1533	000922-62-3	49

```
 9   0.967  6.30 D:\MassHunter\Library\NIST14.L
                 1,5-Hexadiyne                         1031 000628-16-0 53
                 Benzene                               1027 000071-43-2 52
                 Benzene                               1025 000071-43-2 52

10   1.007  8.86 D:\MassHunter\Library\NIST14.L
                 1,3-Hexadien-5-yne                    1036 010420-90-3 43
                 Fumaronitrile                         1022 000764-42-1 43
                 Benzene                               1025 000071-43-2 35

11   1.082  5.55 D:\MassHunter\Library\NIST14.L
                 Cycloheptane                          3352 000291-64-5 59
                 Cyclopentane, 1,3-dimethyl-, cis-     3456 002532-58-3 59
                 Cyclopentane, 1,2-dimethyl-, cis-     3458 001192-18-3 53

12   1.148  6.79 D:\MassHunter\Library\NIST14.L
                 Cyclopentane, 1,3-dimethyl-, cis-     3456 002532-58-3 53
                 1-Nonanol                            21673 000143-08-8 50
                 Cycloheptane                          3352 000291-64-5 50

13   1.243  3.66 D:\MassHunter\Library\NIST14.L
                 Hexane, 3-methyl-                     4043 000589-34-4 56
                 Hexane, 3-methyl-                     4037 000589-34-4 56
                 Hexane, 2,3,4-trimethyl-             12990 000921-47-1 47

14   1.451 18.28 D:\MassHunter\Library\NIST14.L
                 2-Hexenal, (E)-                       3220 006728-26-3 43
                 1H-Imidazole, 4,5-dihydro-2,4-dime    3116 000930-61-0 43
                 thyl-
                 Cyclohexane, methyl-                  3387 000108-87-2 43

15   1.497  0.56 D:\MassHunter\Library\NIST14.L
                 Cyclopentane, 1,2,3-trimethyl-, (1    6959 002613-69-6 58
                 .alpha.,2.alpha.,3.alpha.)-
                 2,3-Dimethyl-1-hexene                 6823 016746-86-4 52
                 Heptane, 3-methylene-                 6825 001632-16-2 52

16   1.539  0.49 D:\MassHunter\Library\NIST14.L
                 Cyclopentane, 1,2,3-trimethyl-, (1    6958 002613-69-6 91
                 .alpha.,2.alpha.,3.alpha.)-
                 Cyclopentane, 1,2,3-trimethyl-, (1    6959 002613-69-6 72
                 .alpha.,2.alpha.,3.alpha.)-
                 Cyclopentane, 1,2,4-trimethyl-, (1    6954 016883-48-0 64
                 .alpha.,2.beta.,4.alpha.)-

17   1.603  0.19 D:\MassHunter\Library\NIST14.L
                 Cyclopentane, 1,2,3-trimethyl-, (1    6956 015890-40-1 91
                 .alpha.,2.alpha.,3.beta.)-
                 Cyclopentane, 1,2,3-trimethyl-        6912 002815-57-8 90
                 Cyclopentane, 1,2,3-trimethyl-, (1    6957 015890-40-1 87
                 .alpha.,2.alpha.,3.beta.)-

18   1.694  0.20 D:\MassHunter\Library\NIST14.L
                 Hexane, 3-ethyl-4-methyl-            13007 003074-77-9 64
                 Carbonic acid, isobutyl 2-ethylhex   93133 1000357-82-9 59
                 yl ester
```

Pk#	RT	Area%	Library/ID	Ref#	CAS#	Qual
			1-Hexene, 3,5,5-trimethyl-	11800	004316-65-8	50
19	1.817	9.37	D:\MassHunter\Library\NIST14.L			
			Toluene	2476	000108-88-3	81
			Toluene	2481	000108-88-3	81
			Toluene	2478	000108-88-3	76
20	1.888	7.82	D:\MassHunter\Library\NIST14.L			
			Toluene	2476	000108-88-3	53
			Toluene	2481	000108-88-3	53
			Toluene	2478	000108-88-3	49
21	1.951	5.55	D:\MassHunter\Library\NIST14.L			
			Cyclohexane, 1,4-dimethyl-, cis-	6927	000624-29-3	49
			Cyclohexane, 1,1-dimethyl-	6867	000590-66-9	47
			Cyclohexane, 1,1-dimethyl-	6871	000590-66-9	47
22	2.015	0.34	D:\MassHunter\Library\NIST14.L			
			Cyclopentane, 1-ethyl-3-methyl-	6916	003726-47-4	52
			Cyclopentane, 1-ethyl-3-methyl-, cis-	6947	002613-66-3	52
			Cyclopentane, 1-ethyl-3-methyl-, trans-	6953	002613-65-2	52
23	2.161	6.40	D:\MassHunter\Library\NIST14.L			
			Octane, 4-methyl-	12942	002216-34-4	47
			Octane, 3,4,5,6-tetramethyl-	40012	062185-21-1	38
			Aziridine, 2,2-dimethyl-	626	002658-24-4	30
24	2.224	0.31	D:\MassHunter\Library\NIST14.L			
			Cyclohexane, 1,4-dimethyl-, trans-	6940	002207-04-7	97
			Cyclohexane, 1,3-dimethyl-, trans-	6938	002207-03-6	95
			Cyclohexane, 1,3-dimethyl-, trans-	6944	002207-03-6	95
25	2.266	0.01	D:\MassHunter\Library\NIST14.L			
			2-Undecene, 4-methyl-	38316	091695-32-8	43
			1-Pentene, 3-methyl-	1495	000760-20-3	38
			Succinic acid, 2-ethylhexyl 3-methylbut-3-en-1-yl ester	157581	1000391-13-7	38
26	2.297	0.01	D:\MassHunter\Library\NIST14.L			
			1-Hexene, 3,5,5-trimethyl-	11800	004316-65-8	53
			Pentadecafluorooctanoic acid, 2-ethylhexyl ester	269071	1000406-80-9	50
			1-Hexene, 3,5,5-trimethyl-	11809	004316-65-8	47
27	2.364	0.01	D:\MassHunter\Library\NIST14.L			
			Octane, 4-methyl-	12942	002216-34-4	50
			Octane	7758	000111-65-9	47
			Decane, 5,6-dimethyl-	39998	001636-43-7	47
28	2.479	0.05	D:\MassHunter\Library\NIST14.L			
			Octane, 2,6-dimethyl-	19673	002051-30-1	50
			Octane, 2,6-dimethyl-	19689	002051-30-1	50
			Dodecane, 2,6,10-trimethyl-	76618	003891-98-3	50
29	2.549	0.38	D:\MassHunter\Library\NIST14.L			

			Cyclohexane, ethyl-	6786	001678-91-7	81
			Cyclohexane, ethyl-	6791	001678-91-7	72
			Cyclohexane, ethyl-	6788	001678-91-7	72
30	2.589	1.46	D:\MassHunter\Library\NIST14.L			
			Cyclohexane, ethyl-	6786	001678-91-7	81
			Cyclohexane, ethyl-	6789	001678-91-7	72
			2-Pentene, 3-ethyl-2-methyl-	6890	019780-67-7	52
31	2.765	0.01	D:\MassHunter\Library\NIST14.L			
			Cyclohexane, 1,1,2-trimethyl-	11817	007094-26-0	72
			Cyclohexane, 1,1,2-trimethyl-	11826	007094-26-0	58
			Cyclooctane, butyl-	38290	016538-93-5	53
32	2.804	0.02	D:\MassHunter\Library\NIST14.L			
			Cyclohexane, 1,1,2-trimethyl-	11817	007094-26-0	64
			Cyclohexane, 1,1,2-trimethyl-	11825	007094-26-0	64
			Cyclohexane, 1,1,2-trimethyl-	11826	007094-26-0	47
33	2.853	0.01	D:\MassHunter\Library\NIST14.L			
			Piperidine	1587	000110-89-4	53
			2-Oxepanone, 7-methyl-	12465	002549-59-9	50
			2-Hexene	1472	000592-43-8	47
34	3.018	0.13	D:\MassHunter\Library\NIST14.L			
			Benzene, 1,3-dimethyl-	5187	000108-38-3	91
			Benzene, 1,3-dimethyl-	5189	000108-38-3	64
			1,3-Cyclopentadiene, 5-(1-methylet<br>hylidene)-	5202	002175-91-9	60
35	3.212	3.73	D:\MassHunter\Library\NIST14.L			
			Benzene, 1,3-dimethyl-	5190	000108-38-3	91
			o-Xylene	5160	000095-47-6	91
			o-Xylene	5168	000095-47-6	91
36	3.278	0.01	D:\MassHunter\Library\NIST14.L			
			3-Heptene, 2,6-dimethyl-	11795	002738-18-3	64
			Cyclopentane, 2-ethyl-1,1-dimethyl	11854	054549-80-3	64
			3-Heptene, 2,6-dimethyl-	11788	002738-18-3	64
37	3.365	0.03	D:\MassHunter\Library\NIST14.L			
			cis-1-Ethyl-3-methyl-cyclohexane	11847	019489-10-2	90
			1-Ethyl-3-methylcyclohexane (c,t)	11849	003728-55-0	83
			Cyclohexane, 1-ethyl-4-methyl-, ci<br>s-	11859	004926-78-7	80
38	3.441	0.00	D:\MassHunter\Library\NIST14.L			
			3,4-Heptadiene	2878	002454-31-1	74
			Cyclohexene, 1-methyl-	2913	000591-49-1	59
			2,4-Hexadiene, 1-chloro-	8460	034632-89-8	53
39	3.557	0.82	D:\MassHunter\Library\NIST14.L			
			Nonane	12939	000111-84-2	70
			Nonane	12937	000111-84-2	70
			Benzene, 1,3-dimethyl-	5186	000108-38-3	59
40	3.645	0.01	D:\MassHunter\Library\NIST14.L			

```
                 1-Trifluoroacetoxy-10-undecene     126138 001675-20-3 47
                 Pentafluoropropionic acid, 10-unde 174562 1000280-07-1 42
                 cenyl ester
                 3-Heptene, 2,6-dimethyl-            11794 002738-18-3 38

41   3.683  0.01 D:\MassHunter\Library\NIST14.L
                 Cyclohexane, 1-ethyl-4-methyl-, ci  11856 004926-78-7 78
                 s-
                 Cyclohexane, 1-ethyl-4-methyl-, ci  11859 004926-78-7 72
                 s-
                 Cyclohexane, 1-ethyl-2-methyl-      11840 003728-54-9 68

42   3.721  0.00 D:\MassHunter\Library\NIST14.L
                 1-Ethyl-3-methylcyclohexane (c,t)   11849 003728-55-0 59
                 cis-1-Ethyl-3-methyl-cyclohexane    11847 019489-10-2 59
                 Cyclohexane, 1-ethyl-4-methyl-, ci  11859 004926-78-7 59
                 s-

43   3.794  0.00 D:\MassHunter\Library\NIST14.L
                 2-Pentene, 3-methyl-, (E)-           1527 000616-12-6 35
                 (2,2-Dimethylcyclobutyl)methylamin   7151 1000195-04-0 28
                 Hexane, 2,4-dimethyl-                7780 000589-43-5 28

44   3.890  0.02 D:\MassHunter\Library\NIST14.L
                 Hexenyl tiglate, 4Z-                49375 1000383-63-6 64
                 (Z),(Z)-2,4-Hexadiene                1238 006108-61-8 49
                 1,3-Butadiene, 2,3-dimethyl-         1270 000513-81-5 47

45   4.065  0.03 D:\MassHunter\Library\NIST14.L
                 Cyclohexane, octyl-                 61872 001795-15-9 59
                 Cyclohexane, octyl-                 61871 001795-15-9 59
                 Cyclohexane, decyl-                 87842 001795-16-0 59

46   4.207  0.01 D:\MassHunter\Library\NIST14.L
                 9-Methyl-Z-10-pentadecen-1-ol      102583 1000131-00-7 47
                 Cyclobutanone, 2-methyl-2-oxiranyl  11423 075314-19-1 27
                 2-Heptene, (E)-                      3359 014686-13-6 22

47   4.350  0.00 D:\MassHunter\Library\NIST14.L
                 3-Heptyne                            2863 002586-89-2 64
                 3-Heptyne                            2860 002586-89-2 56
                 Bicyclo[2.1.0]pentane                 460 000185-94-4 53

48   4.407  0.00 D:\MassHunter\Library\NIST14.L
                 4-Decyne                            17243 002384-86-3 33
                 cis-1-Methyl-2-(2'-propenyl)cyclop   2964 076588-97-1 33
                 ropane
                 Pent-2-ynal                          1174 055136-52-2 32

49   4.450  0.00 D:\MassHunter\Library\NIST14.L
                 Hexane, 2,4-dimethyl-                7782 000589-43-5 25
                 Cyanic acid, 2,2-dimethylpropyl es   7095 001459-44-5 25
                 ter
                 Hexane, 2,4-dimethyl-                7773 000589-43-5 23

50   4.509  0.00 D:\MassHunter\Library\NIST14.L
                 1,15-Pentadecanediol               106112 014722-40-8 38
```

			Cyclohexane, 2-propenyl-	10865	002114-42-3	35
			cis-1-Methyl-2-(2'-propenyl)cyclopropane	2964	076588-97-1	27
51	4.546	0.00	D:\MassHunter\Library\NIST14.L			
			Benzene, propyl-	9586	000103-65-1	87
			Propanedinitrile, (1-methylethenyl)(phenylmethyl)-	61740	069564-95-0	53
			(1,3,3,3-Tetrafluoro-2-trifluoromethyl-propenylsulfanylmethyl)-benzene	162712	075667-95-7	50
52	4.588	0.00	D:\MassHunter\Library\NIST14.L			
			3-Chloropropionic acid, heptadecyl ester	202592	1000283-05-1	38
			7-Octen-1-ol, 2,6-dimethyl-	29222	157615-34-4	37
			2-Heptanol, 5-ethyl-	21701	019780-40-6	37
53	4.656	0.00	D:\MassHunter\Library\NIST14.L			
			Diaziridine, 3-ethyl-3-methyl-	1649	004901-75-1	53
			Heptyl isobutyl carbonate	79587	959068-08-7	43
			Oxetane, 2-methyl-4-propyl-	7723	007045-79-6	37
54	4.720	0.02	D:\MassHunter\Library\NIST14.L			
			Benzene, 1-ethyl-4-methyl-	9610	000622-96-8	93
			Benzene, 1-ethyl-2-methyl-	9608	000611-14-3	93
			Benzene, 1-ethyl-3-methyl-	9609	000620-14-4	91
55	4.851	0.02	D:\MassHunter\Library\NIST14.L			
			Mesitylene	9578	000108-67-8	91
			Benzene, 1,2,4-trimethyl-	9590	000095-63-6	90
			Benzene, 1,2,3-trimethyl-	9595	000526-73-8	87
56	5.007	0.00	D:\MassHunter\Library\NIST14.L			
			Cyclohexane, 1-ethyl-4-methyl-, cis-	11856	004926-78-7	59
			Sulfurous acid, cyclohexylmethyl octadecyl ester	250812	1000309-22-6	59
			Cyclohexane, 1-ethyl-4-methyl-, trans-	11862	006236-88-0	53
57	5.055	0.00	D:\MassHunter\Library\NIST14.L			
			Benzene, 1-ethyl-4-methyl-	9610	000622-96-8	53
			2,4-Nonadiyne	9583	063621-15-8	53
			Benzene, 1-ethyl-2-methyl-	9608	000611-14-3	49
58	5.155	0.00	D:\MassHunter\Library\NIST14.L			
			3,4-Octadiene, 7-methyl-	10867	037050-05-8	83
			Cyclononene	10820	003618-11-9	80
			1,5-Heptadiene	2877	001541-23-7	72
59	5.335	0.06	D:\MassHunter\Library\NIST14.L			
			Benzene, 1-ethyl-3-methyl-	9609	000620-14-4	91
			Mesitylene	9581	000108-67-8	83
			Mesitylene	9578	000108-67-8	64
60	5.758	0.00	D:\MassHunter\Library\NIST14.L			

			Octane, 3-ethyl-	19655	005881-17-4	50
			Cyclopentanol, 3-methyl-	3957	018729-48-1	47
			Decane, 4-methyl-	29367	002847-72-5	47
61	5.886	0.00	D:\MassHunter\Library\NIST14.L			
			2,4-Nonadiyne	9583	063621-15-8	83
			1-Hexen-4-yne, 3-ethylidene-2-methyl-	9632	076003-39-9	42
			Benzene, (1-methylethyl)-	9591	000098-82-8	25
62	5.967	0.00	D:\MassHunter\Library\NIST14.L			
			5-Hepten-2-one, 4,6-dimethyl-	19201	031162-48-8	47
			Cyclohexane, (1-methylpropyl)-	18477	007058-01-7	42
			2-Pentyn-1-ol	1408	006261-22-9	40
63	6.437	0.00	D:\MassHunter\Library\NIST14.L			
			1,3-Pentadiene, (E)-	457	002004-70-8	47
			1,3-Pentadiene, (E)-	455	002004-70-8	47
			1,3-Pentadiene, (Z)-	454	001574-41-0	47
64	6.579	0.00	D:\MassHunter\Library\NIST14.L			
			Benzene, 4-ethyl-1,2-dimethyl-	15224	000934-80-5	72
			Benzene, 1-ethyl-2,4-dimethyl-	15217	000874-41-9	64
			1,3,5-Cycloheptatriene, 3,7,7-trimethyl-	15258	003479-89-8	59
65	7.305	0.00	D:\MassHunter\Library\NIST14.L			
			Oxalic acid, isobutyl octyl ester	118892	1000309-37-3	53
			1-Pentanol, 2-ethyl-4-methyl-	14068	000106-67-2	47
			Oxalic acid, isobutyl nonyl ester	132408	1000309-37-4	40
